# Fundamental Research on the Invention of Breast Support Underwear with a Focus on Women’s Comfort

**DOI:** 10.3390/s23010025

**Published:** 2022-12-20

**Authors:** Minami Isono, Yumiko Tateoka

**Affiliations:** Department of Clinical Nursing, Shiga University of Medical Science, Seta Tsukinowa-cho, Otsu 520-2192, Shiga, Japan

**Keywords:** brassiere, breast support underwear, breast drooping value, breast ptosis pressure measurement system

## Abstract

The purpose of this study was to establish a system for measuring breast underling pressure, evaluate the degree of sustained underling pressure, and verify basic data on the breast lifting distance to improve it. We measured the lifting distance and force at which 24 adult women felt comfortable when their breasts were lifted with an uncovered cloth. The results revealed that the larger the breast size, the greater the pull-up distance and the wider the lifting distance perceived as comfortable. The validity of the measurement method in this study was determined to be useful for the measurement of breast ptosis pressure and breast ptosis position because the measurement was performed at least three times per participant, and the error was small. In the future, we would like to clarify the type of brassiere that supports the breast and gives women a sense of comfort.

## 1. Introduction

During pregnancy, women undergo changes in breast morphology and mammary gland development, although these changes vary among individuals. Estrogen secreted by the placenta during pregnancy primarily stimulates the proliferation and differentiation of the ductal system, whereas prolactin promotes mammary gland growth [1]. After 28 weeks of gestation, the breasts enlarge more markedly, with the top breast increasing by an average of 6.5 cm and the underbust by 10 cm at the time of parturition [2]. In the postpartum period, breast drooping and atrophy are accelerated due to the effects of lactation. Breasts have been shown to deform due to the physical causes of increased mammary tissue weight based on milk production and storage [3]. 

The purpose of wearing bras for perinatal women who bear and nurture children includes the support of growing breasts due to sex hormones during pregnancy and the prevention of minor problems such as stiff shoulders, mastalgia, back pain, and heat during lactation, as well as the rationale for palliative care of breast tension conditions [4]. A study on the physical compatibility and kinetic functionality of bras during lactation stated that non-wire bras are easier to move and less constricting than wire-type bras, but that movement causes nipples to rub against the bra, which can be irritating [5].

Breast movement is generally uncomfortable for women, and bras need to support the breasts. For women with large breasts, bras designed for greater support can reduce force generation and discomfort. It is important to consider the size and volume of the breasts when considering bra design [6]. Asian women have smaller breasts than Western women, but breast support has been shown to be necessary during exercise, regardless of breast size [7].

The breast is subjected to a load of gravity, which stretches the upper skin. In order to reduce the load of gravity on the breast, it is effective to place a sheet inside the bra cup that covers the area around the top of the breast and separates it from the cup [8]. The effectiveness of three-dimensional shape prediction by paper models and finite element analysis of gravity load on the breast has been validated for the bra design [9,10]. It has also been shown that the fit of bra wires to the underbust line can be verified using 3D images [11,12]. All these investigations are general brassiere studies, and no engineering studies have focused on the physiological changes of the breast during the perinatal period. In addition, there have been several studies of bras that women find comfortable, but the relationship between breast-lifting force, distance, and comfort has not been clarified.

Sales related to breast undergarments that are gentle on perinatal women who bear and nurture children have been the focus of underwear manufacturers for the past several years as feminine care products. However, there are no reports addressing basic research on breast underwear from the perspective of sex hormone dynamics during the sexual cycle, pregnancy and lactation, and circulatory dynamics in the breast. In addition, studies on the history of bras in Japan have revealed that no studies have addressed the development of bras as breast care products, suggesting the need for bras that do not interfere with women’s breast changes and provide a sense of comfort [13].

In this research project, as a basic study before tackling the implementation of a support bra for pregnant and nursing women, a breast drooping pressure measurement system was established for general women, and a preliminary study was undertaken to evaluate the degree of sustained drooping pressure and the breast lifting distance to improve it.

## 2. Materials and Methods

### 2.1. Participant Criteria

The participants were 25 healthy adult women with C cups or more who had large breasts and were considered prone to ptosis. The researchers recruited the participants by putting up posters at universities in Shiga Prefecture. The participants had no history of mastopathy or mammary fibrosis and were in the luteal phase of their sexual cycle. Thin women with a BMI of 18 or less were excluded from the study. This study was conducted between October and November 2018.

### 2.2. Overview of the Breast Droop Value Measuring Device

**Figure 1:**
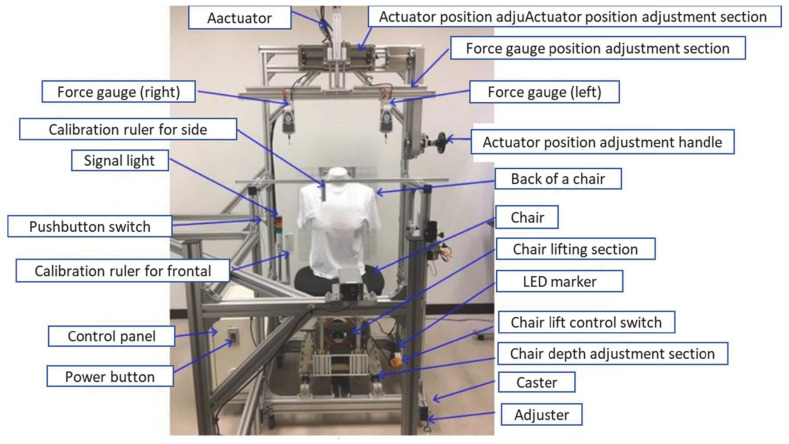
Overview of the breast droop value measuring device.

**Figure 2:**
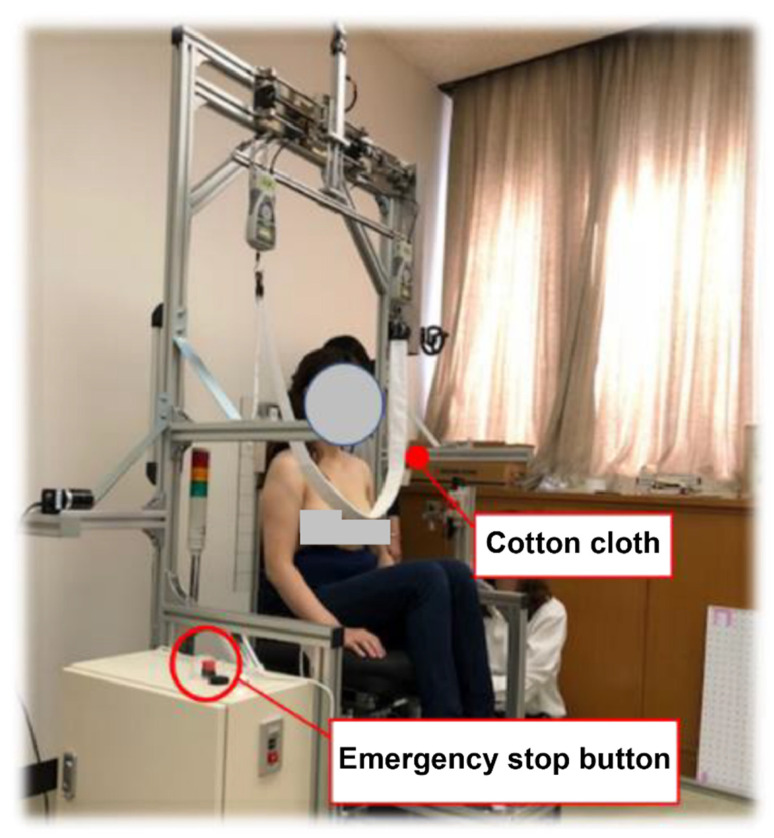
The breast droop value measuring device.

We developed a device to measure the force and distance when the breast was exposed and lifted (Figure 1). The shooting range was from the navel to the nose, and images were taken from the front and right sides with a camera. The right and left breasts were exposed and pulled up with an actuator, and the force was measured using a force gauge (Figure 2). A one-megapixel digital camera was used. Room temperature was set at 25–27 °C.

**Figure 3:**
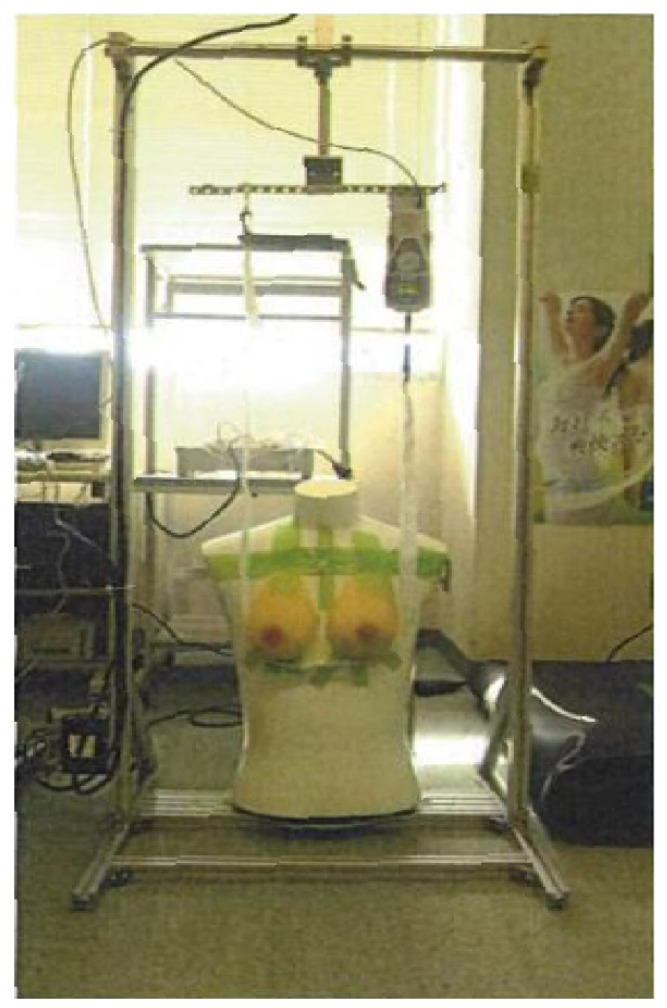
Prototype machine.

The following is an overview of this machine (Figure 3). The machine was 65 cm wide, 64 cm deep, and 143 cm tall. An artificial breast was used to explore the measurement conditions (position adjustment, attachment, pull-up direction, pull-up speed, height of the measurement points, and pull-up interval). The basic conditions for the test were as follows: the weight of the artificial breast was 500 g per side, the exposure was 3 cm, the pull-up speed was 1 mm/s, the interval at which the breast could be lifted vertically was 24 cm, and the lift distance was 10 cm.

### 2.3. Measurement Items and Methods

#### 2.3.1. Measurement Reference Position

The reference position was aligned with the measurement LED marker of the device.

The reference position was adjusted by moving the height and front-back position of the chair while the participant was sitting on a chair so that the marker and reference positions were aligned. The reference position on the participant’s side (human body) was the point at which the underarm intersected the breast when the bleached cloth sheet was placed.

#### 2.3.2. Information about the Bleached Cloth

The width, length, θ, and φ of the bleached cloth were different for each participant, and each item was recorded. The widths of the bleached cloth were 3 and 5 cm. Normally, a width of 3 cm is used. However, 5 cm width was used when the udder could not be lifted sufficiently or when the left and right udders lifted significantly differently. θ and φ were measured with a digital angle meter while the bleached cloth was placed on the udder.

#### 2.3.3. Calibration Location for Video Analysis

**Figure 4:**
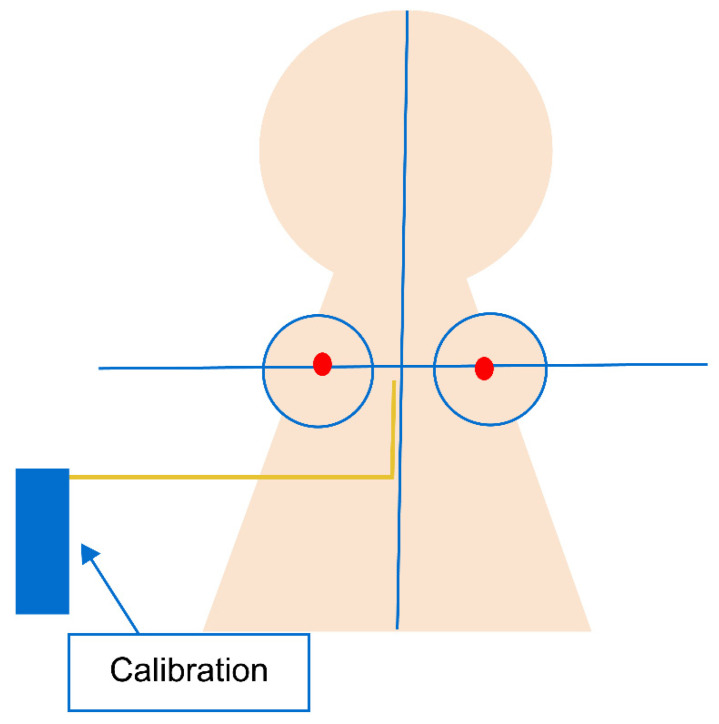
Calibration location for video analysis.

Calibration indicators were used to correctly recognize the distances during the video analysis. The calibration indicator was aligned with the right teat position for more accurate measurement of the teat position change (Figure 4).

#### 2.3.4. Distance between Teats

<actual measurement>

Measurements were taken on the outer side of the right and left teats, with the exposed skin not placed on the udder. Measurements were performed once while sitting on a chair and stretching the back. A Martin pelvimeter (Martin Pelvic Meter MBC Muranaka Medical Instrument) was used for measurement. 

#### 2.3.5. Breast Pull-Up Force

The exposed cloth was passed through the right and left udders and the exposed cloth was pulled up. The speed at which the breast was pulled was 1 mm/s. The data for the pull-up force (N) were obtained while pulling up. The absorbent cloth was pulled up, and the measurement with a force gauge was performed with a breast-drooping value-measuring device. The data of the difference between the left and right force gauge values of 0.5 N or less were obtained three times plus one preliminary time. This is a preliminary study of bra development. Since bras pull up both breasts at the same time, the present study was conducted under conditions in which there was no difference in the pull-up force between the left and right sides.

#### 2.3.6. Range of Comfort during Breast Lift

Data were obtained for the comfort range during breast-lifting. In order to determine the range of comfort, participants were asked to press a button when they felt comfortable when the udder was lifted. The button was then released when the breast was no longer comfortable.

#### 2.3.7. Nipple Position Change

<actual measurement>

The “+” of the LED for teat position measurement was aligned with the right teat, and the scale of the teat position at the start of measurement was recorded. During measurement, the nipple position at the start and end of the range where the breast was lifted and felt comfortable was recorded.

<acquisition of data for video analysis>

The video recording by the front and side cameras started automatically at the same time as the measurement started (start of lifting). The teat position height at the start of the measurement was measured using analysis software.

The start and end of the comfort range were defined as the image when the indicator light was lit yellow (start of comfort) and the image before the indicator light was turned off (end of comfort). Similar to the inter-nipple distance, Frame-DIAS V (DKH Corporation) was used as the video analysis software. From the front camera video, the left and right teat height changes (the teat height at the start of measurement was set to zero), and inter-teat distance changes were analyzed. From the side camera video, the right teat height change (the teat height at the beginning of the measurement was set to zero) was analyzed.

### 2.4. Ethical Considerations

The purpose of this study was explained in writing and in the protocol, and consent was obtained, including permission to obtain the anthropometric information. Participation in this study was voluntary, and consent was obtained after a consent form was signed. In order to avoid the identification of individuals, data were encoded to ensure anonymity. This study was conducted under the Shiga University of Medical Science Collaborative Research Agreement and approved by the Research Ethics Review Committee of Gunze Limited (Project No. 2813146).

### 2.5. Analysis Method

**Figure 5:**
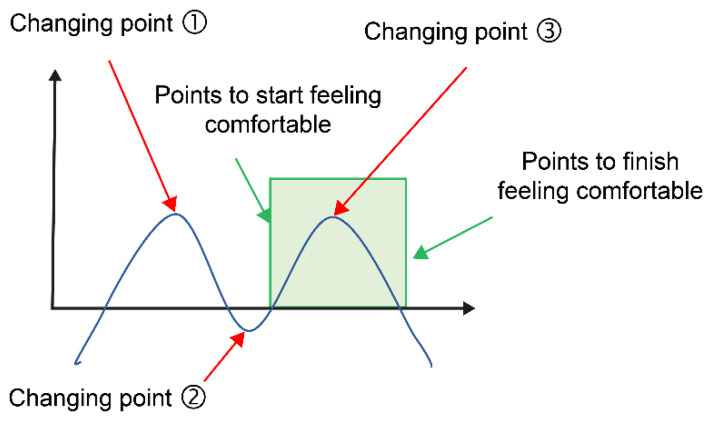
The meanings of the change points.

The sixth-order functions and second-order derivative methods were used for the change points at which the breast was lifted and felt comfortable. The meanings of the change points are shown in Figure 5.

Change point (1): Breast lifting start point
Change point (2): Scientific comfort feeling start point
Change point (3): End point of scientific comfort or best point of scientific comfort.

The graph of the second-order derivative is basically M-shaped or W-shaped, but there are cases where there are two change points or one change point.

In the case of two change points, if any of the change points (1)–(3) can be identified, they are used in the analysis, but if they cannot be identified, they are excluded from the analysis. In the case of one change point, it was excluded from analysis.

IBM SPSS Statistics 26 for Windows was used for the data analysis, and the significance level was set at α < 0.05. The analysis was performed using the Spearman correlation coefficient (Spearman) and t-test.

## 3. Results

### 3.1. Participant Attributes

The 25 study participants had a height of 160.9 ± 5.2 (mean ± SD) cm, a weight of 55.6 ± 7.8 kg, and a BMI of 21.4 ± 2.5. The breast size was C cup in 4 patients, D cup in 9, E cup in 9, F cup in 1, and G cup in 1, and one was unanswered.

### 3.2. Validation of the Breast Drooping Value Measuring Device

Regarding the actuator lifting distance range, a participant with a large breast size and an upper limit of the lifting distance of 10 cm was not sufficient. The lifting distance should be extended to allow for more room for the upper limit. It was necessary to adjust the length of the cloth for each participant because there were large differences between the left and right breasts and individual differences.

### 3.3. Data Exclusion Participants

If a left-right difference of 0.5 N or more was allowed for a force gauge during the measurement, it was excluded from the analysis. Those with insufficient cloth lengths were excluded. As a result, 7 were excluded from the data analysis, and 18 were analyzed.

### 3.4. Results of Measurement Accuracy

#### 3.4.1. Results of Difference between Video Analysis and Actual Measurement

**Figure 6:**
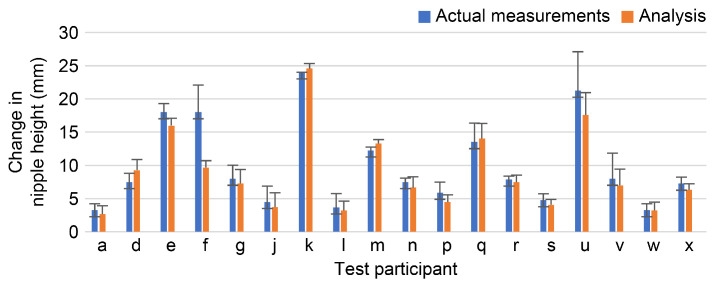
Results of difference between video analysis and actual measurement.

The positional change of the teat during udder raising was measured using video analysis software and compared with the actual measurement data. As a result, the standard deviation of the actual measurement was larger, and the variation in the measurement was smaller when video analysis was used (Figure 6).

#### 3.4.2. Pull-Up Distance Measurement Results

**Figure 7:**
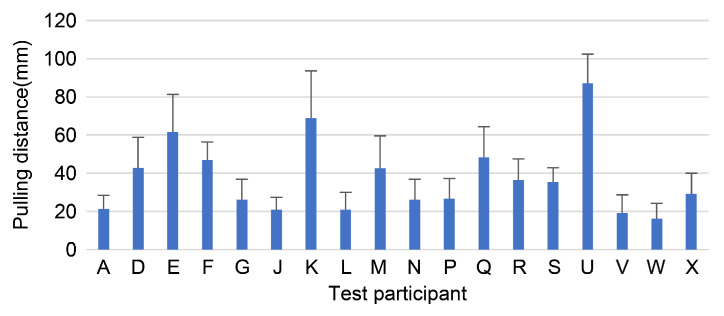
Pull-up distance results.

The mean and standard deviation of the three pull-up distances at the point where each participant felt comfortable during the actual measurements were calculated. Furthermore, errors in the data of the participants were small (Figure 7).

#### 3.4.3. Pull-Up Force Measurement Results

**Figure 8:**
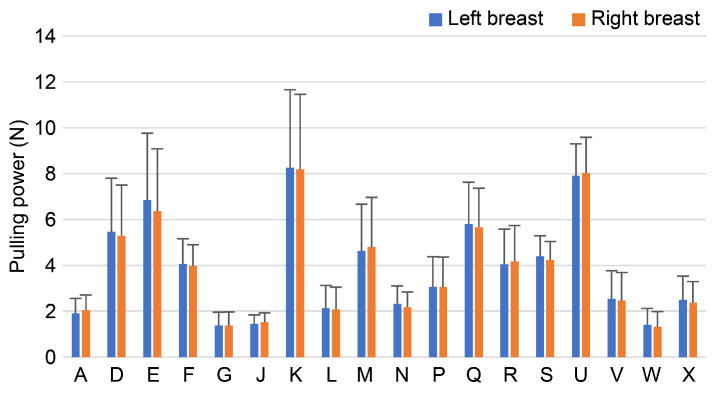
Pull-up force measurement results.

The mean and standard deviation of the three pull-up forces at the point where each participant felt comfortable during the actual measurements were calculated. Errors in the data of the participants were small (Figure 8).

#### 3.4.4. Left-Right Difference in Measured Values

Paired t-tests were performed to determine if there was a difference in the pull-up force between the left and right sides at the point where the force gauge showed a maximum value. No significant differences were found between any of the participants.

A paired *t*-test was performed on the left-right difference in the pull-up force at each change point. There were no significant differences between any of the participants. Therefore, there is no difference in the pull-up forces at each change point.

A paired *t*-test was conducted to determine whether there was a difference in the pull-up force between the left and right breasts within the comfort range. There were no significant differences between any of the participants. Therefore, there was no difference in the pull-up force within the comfort range.

### 3.5. Comparison of Change Points and Sensory Values Related to Breast Pulling Force

**Figure 9:**
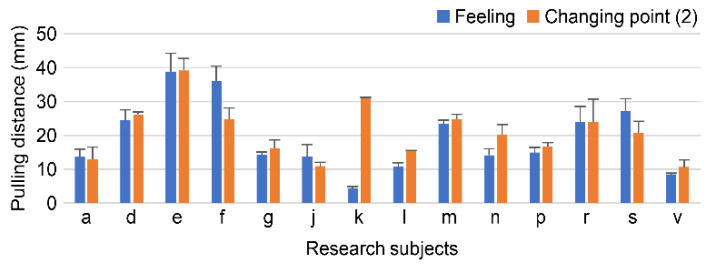
Sensation and point of change when you begin to feel comfortable.

When comparing the values at the point at which the participants began to feel it was easy when the breast was pulled up and the change point (2) (scientific comfort feeling start point), many participants had a difference in values of 10 mm or less (Figure 9). Several participants began to feel sensory ease after the change point (2).

**Figure 10:**
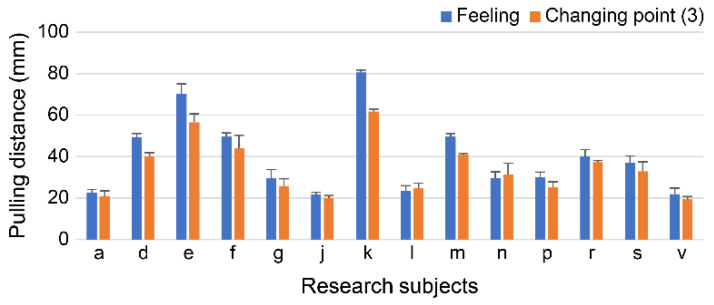
Sensations and points of change when you finish feeling comfortable.

When the values of change point (3) (the endpoint of scientific comfort) were compared with the values at the point when the participant finished feeling comfortable by pulling up the breast, many participants had a difference in values of 10 mm or less (Figure 10). Several participants stopped feeling comfortable (started feeling uncomfortable) after change point (3) (the end point of scientific comfort). If there is a difference between the change point and the sensory value at which they end up feeling easy, the range of feeling easy may be wider.

### 3.6. Correlation with Breast Size

No correlation was found between the pull-up distance (mm) at the start and end of ease and breast size or between the pull-up force (N) at the start and end of ease and breast size (Spearman’s correlation analysis).

### 3.7. Breast Pull-Up Distance and Comfort

The following results were obtained for the pull-up distance at the point at which the breast begins to feel comfortable for each cup size: 14.5 ± 11.1 (m ± SD) mm for cup C, 17.7 ± 10.5 mm for cup D, 21.2 ± 14.6 mm for cup E, 14.0 mm for cup F, and 69.5 mm for cup G.

The following results were obtained for the distance from “the point at which I started feeling comfortable” to “the point at which I finished feeling comfortable” for each breast cup size: the distance was 13.1 ± 9.0 mm for C cup, 15.4 ± 6.6 mm for a D cup, 18.0 ± 11.7 mm for E cup, 15.5 mm for F cup, and 23.4 mm for G cup. Thus, it is clear that as the cup size increases, the pull-up distance perceived as comfortable increases, and the range of perceived comfort is wider.

### 3.8. Breast Pull-Up Force and Feeling of Comfort

The following results were obtained for the pull-up force at the point at which the breast began to feel comfortable for each cup size:

For the right breast, the pull-up force was 1.5 ± 1.5 N for cup C, 1.6 ± 1.0 N for cup D, 2.2 ± 1.8 N for cup E, 1.4 N for cup F, and 6.3 N for cup G. The left breast was 1.5 ± 1.5 N in cup C, 1.6 ± 1.0 N in cup D, 2.1 ± 1.6 N in cup E, 1.4 N in cup F, and 6.3 N in cup G.

The following results were obtained for the change in the pull-up force from the point at which the breast began to feel comfortable to the point at which the breast felt comfortable for each breast cup size:

For the right breast, the pull-up force was 1.4 ± 1.3 N for a C cup, 1.6 ± 1.0 N for a D cup, 2.0 ± 1.7 N for an E cup, 1.2 N for an F cup, and 2.3 N for a G cup. For the left breast, the values were 1.4 ± 1.3 N for cup C, 1.6 ± 1.0 N for cup D, 2.0 ± 1.7 N for cup E, 1.2 N for cup F, and 2.3 N for cup G. It is clear that as the cup size increased, the breast-lifting force increased, and the range of perceived comfort increased.

## 4. Discussion

### 4.1. Establishment of a Breast Ptosis Pressure Measurement System

The following findings were obtained as a result of the verification and validity of the breast ptosis pressure measurement system for adult women:

#### 4.1.1. Specifications of the Breast Droop Pressure Measuring Device

The chair was adjusted to match the participant’s reference position; however, there were no problems with the range of movement (up/down, back/forth). The actuator required a distance of at least 10 cm to pull the exposed cloth up. Since there are large individual differences in breast size and pulling-up distance, the device needs to be improved so that the breast can be pulled up further.

#### 4.1.2. Accuracy of the Udder Pressure Measurement Device

The measurement of nipple position change showed a large variation in the actual measurement, while video analysis was able to measure with a small variation. Therefore, it is clear that accurate measurement is difficult using actual measurements, and video analysis is suitable for accurate measurement.

In order to verify the accuracy of the measurement system, data were collected at least thrice per participant, and the error between the data was verified. The errors were small for both the pull-up distance and pull-up force and were approximately the same among the same participants. There was no difference in the maximum value of the force gauge, the range of force that felt comfortable, or the change in nipple position between the left and right sides. Therefore, the device was found to be useful for measuring the breast ptosis pressure and breast ptosis position.

Okabe et al. reported that bra comfort was related to cup displacement and breast vibration during exercise [14]. Satsumoto et al. stated that a bra with moderate compression of the lower chest position during movement felt more comfortable because it reduced vibrations and shifting [15]. Although this device investigates breast comfort at rest, it is necessary to investigate changes in posture during movement to evaluate comfort.

#### 4.1.3. Variation Points and Sensory Differences

In this study, the interpretation of the three change points calculated using the second-order differentiation of the pull-up force and pull-up distance results was examined. When the values of change point (2) (scientific comfort feeling start point) were compared with the point at which the participant began to feel that it was easy when the breast was pulled up, the difference in values was less than 10 mm for most participants. When the values of change point (3) (the endpoint of scientific comfort) were compared with the point at which the participant felt comfortable when the breast was lifted, many participants had a difference of 10 mm or less in the values. Because the sensory range of feeling easy when lifting the breast and the range calculated from the change point did not deviate significantly, we found the possibility of using the change point as a surrogate index for sensation.

### 4.2. Breast Lift and Comfort

The following findings were obtained from this study on breast-lifting and comfort in the general female population.

It was found that the larger the breast size, the greater the pull-up distance and the wider the distance to be lifted to feel comfortable. This means that as the breast size increases, a bra that can lift over a wider range is needed. Takahashi et al. examined the actual situation of bra size selection among young women and reported that 40% of women chose bras of appropriate size [16]. In addition, in recent years, many women have been wearing camisoles with bra cups because of their convenience, and it is noticeable that many women do not pull their breasts firmly. The results of this study indicate the need for bras that can pull up breasts. In addition, because women increase their breast size by two sizes during pregnancy, the bras they use before pregnancy may not be able to lift the breasts during pregnancy and after childbirth. Therefore, it is necessary for women to review appropriate brands for their own breasts during pregnancy.

It was found that the larger the breast size, the greater the pull-up force and range of perceived comfort. It is inferred that lactating women accumulate, on average, approximately 100 cc of milk per breast. Therefore, it is desirable to support the breast well during the lactation period since more load is placed on the breast, which can cause shoulder stiffness. During lactation, many women experience breast pain and heat during the milk production process. We believe that a comfortable bra for nursing women should consider not only the pull-up distance and pull-up force but also the unique breast condition during the lactation period.

There are wire and non-wire types of bras, as well as various forms of bras. In the future, we would like to clarify what type of brassiere supports breasts and provides women with a sense of comfort. Moreover, we would like to develop bras that are comfortable for women during the perinatal period when the breast shape changes.

### 4.3. Limitations

Mammary tissues undergo repeated changes in proliferation and regression during the menstrual cycle. If the breasts are taut, there may be a difference in comfort when they are lifted. However, the sexual cycle of the participants in this study was unknown. In future studies, the sexual cycle of participants should be ascertained.

In addition, statistical analysis could not be performed when analyzing the breast cup size due to the small sample size. Therefore, it is necessary to set appropriate sample sizes for each breast in future studies.

## Data Availability

Not applicable.
